# Middle-Income Americans and Preparing for Longevity

**DOI:** 10.1093/geroni/igaf122.1987

**Published:** 2025-12-31

**Authors:** Lisa D’Ambrosio, Alexa Balmuth, Lauren Cerino, Adam Felts, Niels Wu

**Affiliations:** Massachusetts Institute of Technology, Cambridge, Massachusetts, United States; Massachusetts Institute of Technology, Cambridge, Massachusetts, United States; Massachusetts Institute of Technology, Cambridge, Massachusetts, United States; Massachusetts Institute of Technology, Cambridge, Massachusetts, United States; Massachusetts Institute of Technology, Cambridge, Massachusetts, United States

## Abstract

With longer lives, people face greater complexity in financial planning and management. While a range of services and products exist to address the needs of wealthy Americans, there remain significant needs and opportunities to support middle-income Americans in planning for their financial futures. To explore this, an online survey was fielded to a nationwide US sample of adults ages 18+ with household incomes between $50,000 and $200,000. Survey items included people’s financial wellbeing, financial information sources, financial knowledge, and demographic characteristics. Results indicated that people were generally positive about being on the path to meeting their future financial goals, but were less confident about being financially prepared for older age. Most thought that they could be doing a better job of planning for their financial futures. Data indicated that everyday Americans valued financial security (88.5%) over moving up the income ladder (11.5%). People reported consulting a range of different sources for financial information and advice, with internet/web searches being the most common source, followed by family and friends and financial professionals. Online sources were least trusted. Participants also reported a relatively high degree of confidence in their financial knowledge and acumen, although within this income band women and people earning less reported were more likely to display lower levels of financial literacy and have less confidence in their financial knowledge. The results point toward opportunities to support everyday Americans around building financial knowledge and connecting them to planning resources.

